# Systematic review (protocol) of clinical effectiveness and models of care of low-resource pulmonary rehabilitation

**DOI:** 10.1038/s41533-019-0122-1

**Published:** 2019-04-05

**Authors:** GM Monsur Habib, Roberto Rabinovich, Kalyani Divgi, Salahuddin Ahmed, Samir Kumar Saha, Sally Singh, Aftab Uddin, Hilary Pinnock

**Affiliations:** 1 Bangladesh Primary Care Respiratory Society, Khulna, Bangladesh; 2 NIHR Global Health Research Unit on Respiratory Health (RESPIRE), Usher Institute of Population Health Sciences and Informatics, The University of Edinburgh, Edinburgh, UK; 3 ELEGI/Colt laboratory, Centre for Inflammation Research, QMRI, The University of Edinburgh and Respiratory Department, Royal Infirmary Edinburgh, Edinburgh, UK; 4 Chest Research Foundation, Pune, India; 5 Johns Hopkins University-Bangladesh, Projahnmo, Dhaka, Bangladesh; 6 Dhaka Shishu Hospital, Dhaka, Bangladesh; 7 Pulmonary and Cardiac Rehabilitation, Department of Respiratory Medicine (Acute Division), University Hospitals of Leicester NHS Trust, Leicester, UK; 8 International Centre for Diarrhoeal Disease Research, Dhaka, Bangladesh

## Abstract

More than half of the people with chronic respiratory diseases (CRDs) live in low- and middle-income countries (LMICs). The increasing disability, reduced productivity, associated anxiety and depression from CRDs result in social isolation and economic hardship for patients and their families. Pulmonary rehabilitation (PR) is a guideline-recommended multidisciplinary and multifaceted intervention that improves the physical and psychological condition of people with CRD. However, PR services are underprovided and uptake is poor in LMICs, especially in low-resourced setting. We aim to systematically assess the effectiveness, applicable components and mode of delivery of PR. We will search MEDLINE, EMBASE, CABI, AMED and CENTRAL from January 1990 using a PICOS search strategy (Population: adults with CRD (including chronic obstructive pulmonary disease, post-tuberculosis, remodelled asthma); Intervention: PR; Comparator: usual care; Outcomes: functional exercise capacity and Health-Related Quality-of-Life; Setting: low-resource settings). Two reviewers will independently screen titles/abstracts and full texts for eligibility and extract data from included papers. We will use the Cochrane Risk-of-Bias tool, rating the quality of evidence using GRADE. We will use narrative synthesis to answer our three objectives: What is the effectiveness of PR in low-resource settings? What components are used in effective studies? What models of service delivery are used? This systematic review will inform the potential impact and practical models of low-resource PR for the betterment of patients with CRDs to improve their substantial health-care burden and address poor quality of life.

## Introduction

The World Health Organisation (WHO) estimates that ‘hundreds of millions’ of people worldwide are affected by chronic respiratory diseases (CRDs), including chronic obstructive pulmonary disease (COPD) (64 million), asthma (235 million), post-tuberculosis (TB) sequelae, bronchiectasis, occupational lung diseases and other often-underdiagnosed conditions responsible for chronic respiratory symptoms.^1^ More than half of those affected are living in low- and middle-income countries (LMICs),^2^ reflecting the influence of major preventable risk factors (especially tobacco smoke, poor environmental air quality, endemic TB) in these countries.^3^ For example, the prevalence of moderate/severe COPD modelled with data from 12 south-east Asian countries has been estimated at 6.3%.^4^ In contrast, a more recent study estimated a COPD prevalence of 10.6% in LMICs globally.^5^ COPD, TB and asthma are all within the top 30 conditions responsible for high rates of disability-adjusted life-years.^6^ CRDs, particularly COPD, are associated with breathlessness and fatigue, which together with muscle dysfunction/wasting contribute to reduced exercise capacity and physical activity levels.^7^ This functional impairment is associated with reduced Health-Related Quality-of-Life (HRQoL), increased exacerbation rates and mortality independent of the degree of airway obstruction.^8,9^ The increasing disability, reduced productivity, associated anxiety and depression result in social isolation and economic hardship for patients and their families.^3^

Pulmonary rehabilitation (PR) is a guideline-recommended multidisciplinary and multifaceted intervention that reduces the burden of chronic respiratory symptoms for people with CRDs.^10,11^ PR is defined as a ‘comprehensive intervention based on a thorough patient assessment followed by patient-tailored therapies that include, but are not limited to, exercise training, education and behaviour change designed to improve the physical and psychological condition of people with CRD and to promote the long-term adherence to health-enhancing behaviours’.^12^ PR improves shortness of breath, exercise tolerance, muscular reconditioning and HRQoL,^13^ and reduces the number^14^ and duration of hospital admissions due to exacerbations.^15^

Although comprehensive programmes of PR offers patient education with provision of self-management plans, psychological therapies to manage anxiety and breathlessness and other elements (potentially including optimisation of treatment in some health-care settings),^16,17^ the cornerstone of PR is an individually tailored physical exercise programme.^18^ The physiological changes produced by aerobic exercise in the muscle contribute to reduced breathlessness and increased endurance exercise capacity.^19^ Although most studies are conducted in well-resourced settings, there is some evidence that less equipped ‘cheaper’ exercise programmes (e.g., walking with increased speed, using resistant rubber bands for exercise) are feasible and may have similar effects to programmes delivered in well-equipped centres.^20^ To be effective, exercise programmes needs to be tailored to an individual in terms of intensity, duration, frequency of sessions, and duration of the total programme.^11^ Sustainability is challenging as stopping exercise after initial success results in loss of benefits over months.^21^

Although the effectiveness of PR in reducing the burden of CRDs is well established,^13,15^ PR services are underprovided even in high-income countries^22–24^ and uptake is poor in LMICs, especially in rural communities.^25^ Lack of trained health professionals to conduct PR, patients’ limited confidence in the effectiveness of PR, and the financial load on the patient and health-care system are barriers to effective programmes.^26,27^ Despite potential cost-effectiveness,^28^ lack of funds for service development precludes implementation in LMICs.^29–31^ There is no systematic review that has rigorously evaluated the effectiveness of a PR service for the (sometimes undifferentiated) range of CRDs seen in LMICs (as opposed to just COPD^32^) designed and implemented within the constraints of resource poor communities. We therefore aimed to systematically search the literature to assess the effectiveness of PR delivered in low-resource settings, the components and the models of care used.

## Objectives

In the context of comprehensive PR (see Table 1 for definition) delivered in low-resource settings, we will:

1. Assess the impact of PR on symptoms, HRQoL, exercise capacity, psychological well-being, rate of exacerbation or hospitalisation, and productivity
2. Identify the components of PR associated with effective low-resource interventions (e.g., minimally equipped exercise programme, type of training, optimisation of cost-effective therapy, education and self-management support, energy conservation training, peer group formation etc.)
3. Describe the service models employed to enable low-cost, sustainable delivery of PR (e.g., duration/frequency of programmes, personnel, venues, equipment, remote access, target population, tele-rehabilitation. etc.)

**Table 1:** PICOS table for the search strategy

Population	Adults with chronic respiratory disease (CRD), including undiagnosed conditions that cause chronic respiratory symptoms. Although most literature from high-income countries is disease specific (typically COPD)^40^ in low-resource settings we anticipate a broader range of diseases and potentially undifferentiated CRD (e.g., COPD, post TB, remodelled asthma, bronchiectasis, interstitial lung disease^41^)
	Comorbidity will not be an exclusion criterion
Intervention	Pulmonary rehabilitation (PR), which includes exercise training (typically aerobic, resistance, and reconditioning,^11^ though local resources and preferences may include other exercise modalities,^42^) and at least one of the following components:^16,43^ patient education, breathing exercises, energy conservation training, peer group interaction, self-management skill development or other recognised PR interventions along with optimisation of pharmacotherapy
	Studies of cardio-pulmonary rehabilitation will be included only if data relating to patients with respiratory disease can be extracted
Comparator	Population who are not given PR—typically ‘usual care’
Outcomes of interest	Primary outcomes will be:
	• Functional exercise capacity (e.g., 6-Minute Walk Test, Incremental Shuttle Walking Test, Endurance Shuttle Walking Test) • Health-Related Quality of Life (HRQoL) (e.g., St. Georges Respiratory Questionnaire (SGRQ), Chronic Respiratory Questionnaire (CRQ)
	Secondary outcomes will be
	• Symptom control: e.g., CCQ; including measures of breathlessness: e.g., MRC Dyspneoa Score, Borg scale • Psychological status, e.g., HADS, PHQ-9 • Health-care burden, e.g., exacerbation rates, hospitalisation etc. • Uptake of the service, completion rates • Adverse effects
Setting	Low-resource settings^44^ typically characterised by lack of funds to cover health-care costs, on individual or societal basis, which leads to one or all of the following:
	• Limited access to medication, equipment, supplies, devices • Less‐developed infrastructure (electrical power, transportation, controlled environment/buildings) • Fewer or less‐trained personnel • Limited access to maintenance and parts • Limited availability of equipment, supplies and medication
	While low-resource settings will often be in LMICs, we will specifically exclude PR delivered in a well-resourced context (e.g., a tertiary care hospital) in an LMIC, and may include interventions in high-income countries if the context is low resource (e.g., remote, deprived community)
Study designs	Randomised control trials (RCTs) and clinical controlled trials

## Methodology

We will follow Cochrane methodology,^33^ and PRISMA reporting standards^34^ to report findings. The review is registered with PROSPERO [ID: CRD42019125326]; any changes to the published protocol will be reported.

### Search strategy

We will develop a comprehensive search strategy using Ovid interface for MEDLINE and EMBASE (Appendix 1), which will be adapted for searching Global Health (CABI), AMED, PubMed, and the Cochrane Database of Controlled Trials (CENTRAL). The strategy will search for ‘Pulmonary Rehabilitation’ AND ‘COPD or other CRD’ AND ‘LMIC or low-resource settings’ from 1990 (the date when global COPD guidelines first recommended PR^35^) with a filter for randomised controlled trials (RCTs) and controlled clinical trials (CCTs). We will undertake forward citation on included studies, and check reference lists for relevant studies. We will search clinical trial registers for ongoing trials and search for publications of any abstracts that we identify. We do not plan to undertake hand searching unless we find a journal that regularly publishes relevant PR papers. We are interested in studies from LMICs and will, therefore, not impose a language restriction, aiming to arrange translation if the English abstract suggests it may be relevant.^36^

We will export all the searched literature to EndNote for de-duplication, screening processes and overall data management.

### Selection process

Our PICOS strategy is detailed in Table 1. In summary, we will select papers that compare a PR intervention delivered in a low-resource setting for people with COPD/other CRDs with usual care. Following training, two reviewers (M.H. and S.A. or K.D.) will screen titles and abstracts and identify potentially eligible studies. Disagreements will be resolved by discussion between reviewers, involving H.P. or R.R. as necessary. After the retrieval of the full-text of potentially eligible studies, two reviewers (M.H. and S.A. or K.D.) will independently screen the studies against the selection criteria (see Table 1). Disagreements will be resolved by discussion with the team (H.P., R.R., S.S. and A.U.) to determine rules for operationalising the inclusion/exclusion criteria. If anything, remains unclear, the authors will be contacted and if this fails, the study will be listed as ‘potentially relevant study’. We will report all the processes in a PRISMA flow diagram,^34^ and tabulate excluded full-text papers with reasons for exclusion.

### Outcome measurement

Our primary outcomes will be functional exercise capacity and HRQoL. For details and description of secondary outcomes see Table 1.

### Data management and extraction

Based on the Cochrane EPOC guidance,^37^ we will develop a customised data extraction form, which will be piloted to ensure its easy and consistent interpretation and capture of all relevant information, including PICOS criteria, definitions used and outcome measurements. We will collate multiple reports of the same study so that each study, rather than each report, is the unit of interest in the review.

### Risk of bias assessment

Two reviewers (M.H. and S.A. or K.D.) will independently assess the methodological quality of all included articles according to the Cochrane Risk of Bias’ tool.^33^ We will assess the papers for selection, performance, detection, attrition, reporting and other sources of bias, and assess the overall risk of bias. A summary of the assessment will be recorded and tabulated with the overall judgement.

### Data analysis

The analysis will address our three objectives as follows:

1. Effectiveness of PR in low-resource settings: On the basis of our initial scoping, we anticipate that our included studies will have substantial clinical, methodological and statistical heterogeneity, and meta-analysis will not be appropriate. If so, we will conduct a narrative synthesis potentially using graphical techniques (e.g., Harvest plots^38^) to illustrate the key outcomes and their relationships.
2. Components used in effective studies: We will identify the components included in the PR service (exercise training and other components^11^).
3. Models of care used in the PR interventions. We will describe the models of care used, including personnel and their training, venue and equipment available, number and frequency of sessions, use of telehealth and strategies for sustainability.

### Interpreting the findings

We will use the GRADE approach^39^ to rate the quality of evidence for the primary outcomes and the important secondary outcomes (listed in Table 1).

### Dissemination

We will present the findings of the systematic review at national and international conferences, and publish in a peer-reviewed journal. In addition, we will use the researchers’ professional networks and the innovative dissemination strategies of the NIHR Global Health Research Unit on Respiratory Health (RESPIRE), including social media.

## Conclusion

PR is an integral component of the management of people with CRDs, particularly for patients with COPD. This is a major challenge for LMICs who bear a disproportionate burden of CRD but without the resources to develop effective PR services. There is an unmet need to implement PR in these countries with a model that is effective, deliverable and sustainable in low-resource settings. Indeed, locally delivered low-cost PR may be more sustainable in some health-care economies than unaffordable long-term medication. The findings of this review may inform the potential impact and practical models of low-resource PR for the betterment of patients with CRDs in order to improve their substantial health-care burden, and address poor quality of life.

## Supplementary information

Appendix 1

## Supplementary information

**Supplementary information** accompanies the paper on the *npj Primary Care Respiratory Medicine* website (10.1038/s41533-019-0122-1).
