# Supplementary material for: Systematic review (protocol) of clinical effectiveness and models of care of low-resource pulmonary rehabilitation
Source: NPJ Prim Care Respir Med. 2019 Apr 5;29:10. doi: 10.1038/s41533-019-0122-1 (PMC6450955; doi:10.1038/s41533-019-0122-1)
Supplement: Supplementary file 1 — Appendix 1 [file 41533_2019_122_MOESM1_ESM.docx]

| [# ▲](http://ovidsp.tx.ovid.com.ezproxy.is.ed.ac.uk/sp-3.31.1b/ovidweb.cgi?&S=BGGDFPAGCIDDKCCINCEKCEMCNIKOAA00&Sort+Sets=descending) | **Searches of MEDLINE on 28 Oct. 18** | | **Results** |
| --- | --- | --- | --- |
| 1 | exp Lung Diseases, Obstructive/ | | 197888 |
| 2 | pulmonary disease, chronic obstructive.mp. or exp Pulmonary Disease, Chronic Obstructive/ | | 49651 |
| 3 | emphysema$.mp. | | 31795 |
| 4 | (Chronic$ adj3 bronchiti$).mp. | | 10714 |
| 5 | (obstruct$ adj3 (pulmonary or lung$ or airway$ or airflow$ or bronch$ or respirat$)).mp. | | 98543 |
| 6 | COPD.mp. | | 34381 |
| 7 | COAD.mp. | | 222 |
| 8 | COBD.mp. | | 12 |
| 9 | AECB.mp. | | 210 |
| 10 | (obstruct* adj3 (pulmonary or lung* or airway* or airflow* or bronch* or respirat*)).mp. | | 98543 |
| 11 | AECOPD.mp. | | 630 |
| 12 | exp chronic bronchitis/ | | 1688 |
| 13 | 1 or 2 or 3 or 4 or 5 or 6 or 7 or 8 or 9 or 10 or 11 or 12 | | 252449 |
| 14 | REHABILITATION.mp. or exp REHABILITATION/ | | 459193 |
| 15 | Respiratory Therapy.mp. or exp Respiratory Therapy/ | | 106770 |
| 16 | Physical Therapy Modalities.mp. or exp Physical Therapy Modalities/ | | 138907 |
| 17 | (rehabilitat* or fitness* or exercis* or train* or physiotherap* or (physical* adj therap*)).mp. | | 968222 |
| 18 | 14 or 15 or 16 or 17 | | 1197761 |
| 19 | (Africa or Asia or Caribbean or "West Indies" or "South America" or "Latin America" or "Central America").mp. | | 220128 |
| 20 | (Afghanistan or Albania or Algeria or Angola or Antigua or Barbuda or Argentina or Armenia or Armenian or Aruba or Azerbaijan or Bahrain or Bangladesh or Barbados or Benin or Byelarus or Byelorussian or Belarus or Belorussian or Belorussia or Belize or Bhutan or Bolivia or Bosnia or Herzegovina or Hercegovina or Botswana or Brasil or Brazil or Bulgaria or "Burkina Faso" or "Burkina Fasso" or "Upper Volta" or Burundi or Urundi or Cambodia or "Khmer Republic" or Kampuchea or Cameroon or Cameroons or Cameron or Camerons or "Cape Verde" or "Central African Republic" or Chad or Chile or China or Colombia or Comoros or "Comoro Islands" or Comores or Mayotte or Congo or Zaire or "Costa Rica" or "Cote d'Ivoire" or "Ivory Coast" or Croatia or Cuba or Cyprus or Czechoslovakia or "Czech Republic" or Slovakia or "Slovak Republic").mp. | | 436693 |
| 21 | (Djibouti or "French Somaliland" or Dominica or "Dominican Republic" or "East Timor" or "East Timur" or "Timor Leste" or Ecuador or Egypt or "United Arab Republic" or "El Salvador" or Eritrea or Estonia or Ethiopia or Fiji or Gabon or "Gabonese Republic" or Gambia or Gaza or Georgia or Georgian or Ghana or "Gold Coast" or Greece or Grenada or Guatemala or Guinea or Guam or Guiana or Guyana or Haiti or Honduras or Hungary or India or Maldives or Indonesia or Iran or Iraq or "Isle of Man" or Jamaica or Jordan or Kazakhstan or Kazakh or Kenya or Kiribati or Korea or Kosovo or Kyrgyzstan or Kirghizia or "Kyrgyz Republic" or Kirghiz or Kirgizstan or "Lao PDR" or Laos or Latvia or Lebanon or Lesotho or Basutoland or Liberia or Libya or Lithuania).mp. | | 516935 |
| 22 | (Macedonia or Madagascar or "Malagasy Republic" or Malaysia or Malaya or Malay or Sabah or Sarawak or Malawi or Nyasaland or Mali or Malta or "Marshall Islands" or Mauritania or Mauritius or "Agalega Islands" or Mexico or Micronesia or "Middle East" or Moldova or Moldovia or Moldovian or Mongolia or Montenegro or Morocco or Ifni or Mozambique or Myanmar or Myanma or Burma or Namibia or Nepal or "Netherlands Antilles" or "New Caledonia" or Nicaragua or Niger or Nigeria or "Northern Mariana Islands" or Oman or Muscat or Pakistan or Palau or Palestine or Panama or Paraguay or Peru or Philippines or Philipines or Phillipines or Phillippines or Poland or Portugal or "Puerto Rico").mp. | | 274812 |
| 23 | (Romania or Rumania or Roumania or Russia or Russian or Rwanda or Ruanda or "Saint Kitts" or "St Kitts" or Nevis or "Saint Lucia" or "St Lucia" or "Saint Vincent" or "St Vincent" or Grenadines or Samoa or "Samoan Islands" or "Navigator Island" or "Navigator Islands" or "Sao Tome" or "Saudi Arabia" or Senegal or Serbia or Montenegro or Seychelles or "Sierra Leone" or Slovenia or "Sri Lanka" or Ceylon or "Solomon Islands" or Somalia or Sudan or Suriname or Surinam or Swaziland or Syria or Tajikistan or Tadzhikistan or Tadjikistan or Tadzhik or Tanzania or Thailand or Togo or "Togolese Republic" or Tonga or Trinidad or Tobago or Tunisia or Turkey or Turkmenistan or Turkmen or Uganda or Ukraine or Uruguay or USSR or "Soviet Union" or "Union of Soviet Socialist Republics" or Uzbekistan or Uzbek or Vanuatu or "New Hebrides" or Venezuela or Vietnam or "Viet Nam" or "West Bank" or Yemen or Yugoslavia or Zambia or Zimbabwe or Rhodesia).mp. | | 328981 |
| 24 | ((developing or "less* developed" or "under developed" or underdeveloped or "middle income" or "low* income" or underserved or "under served" or deprived or poor*) adj (countr* or nation* or population* or world)).mp. | | 127486 |
| 25 | ((developing or "less* developed" or "under developed" or underdeveloped or "middle income" or "low* income") adj (economy or economies)).mp. | | 351 |
| 26 | (low* adj (GDP or GNP or "gross domestic" or "gross national")).mp. | | 203 |
| 27 | (low adj3 middle adj3 countr*).mp. | | 8403 |
| 28 | (LMIC or LMICs or "third world" or "LAMI country" or "LAMI countries").mp. | | 4711 |
| 29 | ("transitional country" or "transitional countries").mp. | | 131 |
| 30 | (setting* adj2 (resource* or poor* or constrain* or low*)).mp. | | 12989 |
| 31 | 19 or 20 or 21 or 22 or 23 or 24 or 25 or 26 or 27 or 28 or 29 or 30 | | 1629217 |
| 32 | 13 and 18 and 31 | | 1370 |
|  | |  |  |
